# The Influence of Beer, Tobacco, Etc., upon Digestion

**Published:** 1886-10

**Authors:** 


### The Influence of Alcohol, of Beer, of Black Coffee, Tobacco, Salt, and Alum upon Digestion.

Dr. C. Bikfalvi, of Klausenburg, reports, according to the *Deutsche Medicinal Zeitung*, upon a series of experimental studies of digestion to the following effect:

1. Alcohol, even in small quantities, arrests the digestive processes. The digestion of albuminates is arrested more than the transformation of dextrine to grape sugar. Gastric juice, with 20 per cent. of alcohol, digests six to seven times smaller quantities than the normal secretion. This is explained by the precipitation of pepsin by the alcohol.

2. Beer does not promote digestion. It appears that this is due, not so much to its alcohol as to the presence of large quantities of neutral salts that bind the free acid of the gastric secretion. If a few drops of hydrochloric acid are added, the beer no longer inhibits.

3. Wine in small quantities appears to promote digestion; in larger quantities its action is that of alcohol.

4. Black coffee, also, when taken in small quantities, stimulates the digestive functions; large quantities act unfavorably.

5. Moderate smoking does not alter digestion. Excessive smoking, however, is of bad influence, because the tobacco derivatives—alkaline reaction of nicotine—neutralizes the gastric juice.

6. Small quantities of salt are conducive to the processes under consideration. Large quantities arrest them, probably by hindering the swelling of the food.

7. Alum is decidedly injurious to digestion. Even the changes of dextrine is retarded by small quantities. It is used by some bakers in bread; this should not be tolerated for the above given reasons.

